# Salutogenesis as a theoretical framework for psychosocial rehabilitation: the case of the Clubhouse model

**DOI:** 10.1080/17482631.2020.1748942

**Published:** 2020-04-06

**Authors:** Orsolya Reka Fekete, Liv Grethe Kinn, Torill M. B. Larsen, Eva Langeland

**Affiliations:** a Department of Welfare and Participation, Faculty of Health and Social Sciences, Western Norway University of Applied Sciences and Department of Health Promotion and Development Faculty of Psychology, University of Bergen, Bergen, Norway; b Department of Welfare and Participation, Faculty of Health and Social Sciences, Western Norway University of Applied Sciences, Bergen, Norway; c Department of Health Promotion and Development Faculty of Psychology, University of Bergen, Bergen, Norway; d Department of Health and Caring Sciences, Faculty of Health and Social Sciences, Western Norway University of Applied Sciences, Bergen, Norway

**Keywords:** Clubhouse model, mental health promotion, mental illness, psychosocial rehabilitation, salutogenesis

## Abstract

**Purpose**: This study explored whether the holistic theory of salutogenesis may be a suitable theoretical framework for the Clubhouse model of psychosocial rehabilitation, a pioneer among psychosocial rehabilitation programmes.

**Methods**: A systematic examination of elements of the Clubhouse model, as prescribed by the Clubhouse standards, was performed within the context of the theory of salutogenesis including its basic salutogenic orientation and the main concepts of sense of coherence and resistance resources.

**Results**: We found that several standards and practices within the Clubhouse model can be understood as applications of salutogenesis. We have hypothesized that the Clubhouse model promotes peoples’ sense of coherence and mental health. However, our investigation also showed that, to enhance the recovery of Clubhouse members, more explicitly incorporating some salutogenic principles, such as “appropriate challenges” and “active adaptation as the ideal in treatment”, may benefit Clubhouse practice.

**Conclusions**: The Clubhouse model of psychosocial rehabilitation is very consistent with the salutogenic orientation and main salutogenic concepts. The present study suggests that salutogenesis may be a suitable theoretical framework for the Clubhouse model and possibly in the psychosocial rehabilitation field in general.

## Introduction

The past two decades have seen major changes in the mental health-care field. For example, the World Health Organization (2013) introduced a new definition of mental illness, which acknowledged that it is a complex psychosocial issue beyond being a medical condition. In addition, recovery orientation has emerged as the mainstream policy in mental health care around the world (Anthony & Mizock, 2014; Davidson et al., 2007; Jacobson & Curtis, 2000; Pilgrim, 2008; Ramon et al., 2007; World Health Organization, 2013) as well as in Norway (Ministry of Labour & Ministry of Health and Care Services., 2013; Norwegian Directorate of Social Services and Health, 2005). Community-based psychosocial rehabilitation (PSR) services have become increasingly important in mental health care (Farkas, 2006).

PSR is a multifaceted field that aims to contribute to the recovery of people with persistent mental illness by “enhancing their role functioning in a role valued by society and selected by the individual” (Farkas & Anthony, 2010, p. 116). Even though PSR includes many types of services, it has well-defined principles and a value base (Farkas, 2006; Farkas & Anthony, 2010; Rössler, 2006). These include empowerment, voluntarism, autonomy, partnership, the importance of hope, a focus on strengths and interests versus illness and limitations, and a results orientation. Different PSR services targeting different outcomes generally use similar techniques, such as skills training to improve role performance, providing support to improve role success, and advocacy to increase societal opportunities (Farkas, 2006; Farkas et al., 2007; Rössler, 2006). It is possible to provide a general description of the process of any PSR intervention (Farkas & Anthony, 2010), which comprises three phases: choosing or designating a goal; getting, which means taking steps to reach the goal; and keeping, meaning to maintain the achievement.

Despite the well-defined universal targets, values, principles, and techniques, the evaluation of PSR interventions has been somewhat elusive given the multifaceted nature of PSR (Farkas & Anthony, 2010; Farkas et al., 2007). For instance, within the population of people with mental illness, PSR deals with several distinct target groups, all of whom require different approaches and sets of techniques (Farkas et al., 2007). Moreover, different interventions target different outcomes, even though some of them provide comprehensive services (Rössler, 2006). Because PSR services operate in different locations and in different cultures, the picture becomes even more complex and has resulted in small sample sizes for research. Consequently, randomized controlled trial studies and other comparative analyses are difficult to conduct, meaning that research and evaluation have focused predominantly on individual interventions. Therefore, establishing a complete picture of PSR services is difficult, but necessary, for example, by developing comprehensive, complementary, and well-functioning PSR services without overlap to spare resources in a particular area.

As a groundbreaking programme in the late 1940s, the Clubhouse model was a pioneering PSR intervention that contributed greatly to the development of the principles guiding the field (Anthony & Liberman, 1986; Cnaan et al., 1988). Originally developed in New York City, the model is considered to be a well-established recovery- and consumer-oriented intervention (Anthony & Liberman, 1986; Clubhouse International, 2018; Farkas et al., 2007; Stoffel, 2011). The Clubhouse model offers participation in meaningful activities to promote the recovery of people with mental illness by targeting a wide range of outcomes, including but not limited to social, vocational, housing, and citizenship issues (Cnaan et al., 1988; C. McKay et al., 2016). Today, there are some 300 Clubhouses around the world (Clubhouse International, 2017). Ensuring their adherence to the Clubhouse model is a set of 37 standards, the International Standards for Clubhouse Programs, (referred to as “the standards” in this paper) (Clubhouse International, 2018).

The need to understand the active ingredients of the Clubhouse model, or how it achieves its outcomes, has been identified in the Clubhouse literature (Mowbray et al., 2006; Tanaka & Davidson, 2015a, 2015b). Some have suggested that a theory might help this endeavour. For instance, Raeburn et al. (2015) argued that self-determination theory might help to understand better the workings of the Clubhouse model. However, another team (Mutschler et al., 2018) took a more empirical approach by developing a realist theory for the model. The present paper explores a broader and more generic direction by examining whether the theory of salutogenesis might be a suitable theory for the Clubhouse model.

A theoretical framework is a crucial element when researching and evaluating programs (Leavy, 2014). As Langeland et al. (2007, p. 276) have argued, “An intervention is not ready to be evaluated unless the theoretical basis of the intervention has been developed and carried out.” In addition, a theoretical framework “can illuminate areas that might not otherwise be visible” regarding a subject matter (Taylor, 2004, p. 633) and can foster association with existing bodies of research (Chambliss & Schutt, 2006).

The theory of salutogenesis provides a generic understanding of how coping, defined as a sense of coherence (SOC), and well-being may be created. The theory is used in several fields, such as nursing and mental health care (Eriksson & Lindström, 2006; Griffiths, 2009; Langeland & Vinje, 2016). Salutogenesis has also been suggested as a suitable framework for public health development (Lindström & Eriksson, 2006), to guide health promotion (A. Antonovsky, 1996; García-Moya & Morgan, 2017), mental health rehabilitation (Griffiths, 2009), and mental health promotion (Langeland & Vinje, 2013). The main purpose of the present study is to explore how salutogenesis might provide also a theoretical framework for the psychosocial Clubhouse rehabilitation model.

## Salutogenic theory

Contrary to the biomedical model, which considers a person only in terms of their illness, salutogenesis offers a positive approach to health that is outlined by five basic assumptions (A. Antonovsky, 1979, 1987). First, health is defined as a continuum based on an understanding that although ill, a person still has healthy attributes to build on and is, therefore, in a state between health breakdown (dis-ease) and full health (ease). Second, it is the ’story of the person‘ as a whole that matters, in a holistic sense, rather than the illness focus of the medical approach. Third, “health-promoting (salutary) factors” or opportunities must be the centre of attention, rather than the pathogens or risk factors. Fourth, tension and strain are potentially health promoting rather than a ubiquitous evil to fight. Fifth, active adaptation is the ideal in treatment instead of assuming a “right treatment based on the right diagnosis” approach. Accordingly, salutogenesis focuses on the person in his/her entirety by interacting with both internal and external environments.

The main concept of the salutogenic model, determining a person’s ability to stay well, is the SOC (A. Antonovsky, 1979, 1987). The SOC is defined as a global orientation that expresses the extent to which one can make sense of a challenge and address it successfully if it is deemed worth dealing with. The core components defining the SOC are comprehensibility, manageability, and meaningfulness (A. Antonovsky, 1979, 1987). According to Antonovsky, the third component, meaningfulness, is the most important because it is an emotional–motivational entity and plays a crucial role in shaping the outcome by determining whether something matters enough for the person to deal with. To maintain or increase the level of meaningfulness, Antonovsky suggested investment in four areas fundamental to life: main activity, inner feelings, social relationships, and existential issues (Langeland & Vinje, 2016, p. 301).

According to salutogenic theory, resistance resources (RRs) are another important determinant of well-being (A. Antonovsky, 1979, 1987). Based on their scope of usefulness, RRs can be divided into two groups: generalized resistance resources (GRRs) with a “wide-ranging utility” and specific resistance resources (SRRs) that have a “situation-specific utility” (Mittelmark et al., 2016, p. 71). A GRR is defined as “any characteristic of the person, the group, or the environment that can facilitate effective tension management” (Vinje et al., 2017, p. 29).A GRR is also considered to be a consistent life experience that has a role in shaping an outcome and poses an appropriate challenge. It can also play a role in facilitating the SOC by promoting the development of any of its core components (Idan et al., 2016).For example, a GRR is an individual’s social network that the person can rely on in various situations. An SRR may be an emergency phone number for requesting an ambulance in case of an accident (Mittelmark et al., 2016, p. 71).

The ability to use one’s RRs determines whether a challenge is “appropriate.” An appropriate challenge is defined as an occurrence that is neither too easy nor too difficult to overcome. In salutogenic terms, a challenge has underload/overload balance (Idan et al., 2016). Although RRs help in the development of the SOC, having the experience of a strong SOC may help to shape a person’s experiences (or RRs) in return (A. Antonovsky, 1979, 1987). However, a lack of resources, called resistance deficits (RDs), weakens the SOC. In the continuum model, RDs, which represent the lack of resources to combat the challenges of life, are at the opposite end of the scale from RRs (A. Antonovsky, 1987). Consequently, an RR might be anything that promotes the SOC, whereas an RD represents a lack of resources that can weaken the SOC (A. Antonovsky, 1987).

**Figure 1.**
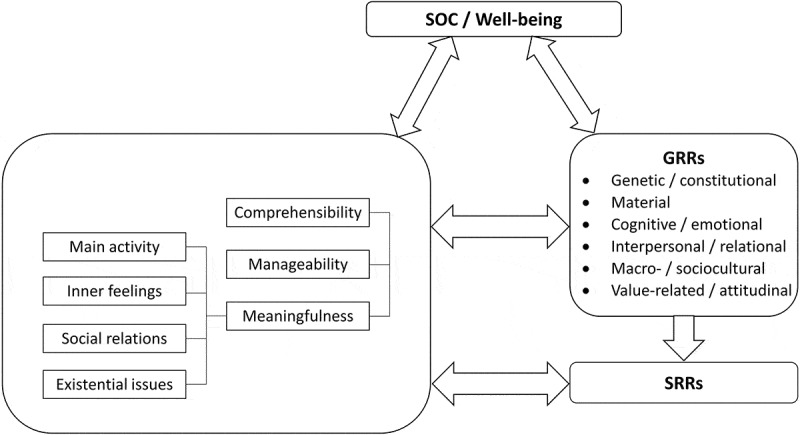
The interplay between well-being, sense of coherence (SOC) and its core components, and the different types of resistance resources (RRs)

Figure 1 illustrates the complex reciprocal interaction between well-being, SOC and its core components, and RRs. This figure includes a list of some of the major types of GRRs based on the works of A. Antonovsky (1987), Sullivan (1989), and Langeland et al. (2007).

In terms of the different types of RRs that can promote SOC and well-being, Antonovsky emphasized that social support and self-identity are the most crucial coping resources (Langeland et al., 2007). Langeland and Vinje (2016) elaborated that a well-functioning social network may be a source of several further assets for coping, such as availability of help, guidance, alliance, and reassurance among others. At the same time, being conscious of one’s identity is helpful for developing a self-appropriate position within this social network and may contribute to a realistic insight into one’s social capabilities. Furthermore, the value-related content of identity, self-esteem, is an important factor in terms of meaningfulness as the motivation for coping by translating to the notion “I am worthy of dealing with this challenge.”

## The Clubhouse model

The Clubhouse model has developed through a cumulative and pragmatic learning-from-experience approach (Anderson, 1998; Propst, 1997), which has resulted in the formulation of the (current) 37 standards (Clubhouse International, 2018). The standards are organized into eight core domains: membership, relationships, space, work-ordered day, employment, education, functions and funding, and governance and administration (Clubhouse International, 2018).

Central to the model is that it operates as a Clubhouse; therefore, those who participate are referred to as members instead of users, clients, or patients (Propst, 1997), which results in an egalitarian structure (C. McKay et al., 2016; Tanaka & Davidson, 2015b). Membership has a low threshold and the only criterion is to have a history of mental illness (Clubhouse International, 2018, p. 2). It is also stipulated that membership is voluntary and without any time limit (Clubhouse International, 2018, p. 1), which provides stable, long-term support, which has been shown to have a greater impact on long-term rehabilitation outcomes for people with mental illness than short-term early intervention (Mowbray et al., 2006).

Members’ rights are balanced with their responsibilities. The section on relationships in the standards stipulates that members are expected to actively take part in the running of the Clubhouse, “side-by-side” with the generalist staff; Clubhouses are intended to be understaffed so they cannot be run without member participation (Clubhouse International, 2018, p. 9). However, Tanaka and Davidson (2015b) observed that all members are treated as being contributors to the community regardless of their level of participation in everyday tasks. Staff are expected to encourage member participation by working together as equals (Clubhouse International, 2018, pp. 10–11). Studies have shown that both reciprocity in relationships and a positive therapeutic alliance foster recovery from mental illness (Fekete et al., 2020; Kidd et al., 2017; Roth, 2017; Tanaka & Davidson, 2015b). Furthermore, Staples and Stein (2008) describe the Clubhouse model as a unique hybrid of self-help and staff and peer support that provides multiple levels of support to members within the Clubhouse.

The section on space regulates three main areas. First, the Clubhouse must be an individual legal entity (Clubhouse International, 2018, p. 12). Second, the Clubhouse must be separate from any treatment facility or other programs, in addition to providing a place that conveys dignity and professionalism (Clubhouse International, 2018, p. 13). Third, the egalitarian structure in the model is emphasized by providing standards-bound rights to equal access and equal opportunities in using the Clubhouse for both members and staff (Clubhouse International, 2018, p. 14).

Work (employment) is both a tool and a goal within the Clubhouse model (C. E. McKay et al., 2006; Kinn et al., 2018a; C. McKay et al., 2016; Norman, 2006). Work as a tool emerges from the section dealing with the work-ordered day (WOD), which denotes that (a) the WOD parallels typical working hours or the workday, (b) the WOD provides the framework for all Clubhouse-related activities, and (c) all work generated within the Clubhouse must serve the maintenance or development of the Clubhouse facilities and community. Setting work as the main focus may also help to focus on members’ strengths, or the things they can contribute to the community, which in return fosters a sense of mastery and achievement for the individual (Tanaka & Davidson, 2015a). Studies have shown that the WOD helps members’ sense of autonomy and promotes their social development (Tanaka & Davidson, 2015a), and provides a framework for the “turning points” (i.e., major positive changes) in members’ lives (Norman, 2006).

Work as a goal emerges from the section on employment within the standards (Clubhouse International, 2018), which state clearly that the Clubhouse is intended to “enable its members to return to paid work” (Clubhouse International, 2018, p. 21) and to offer a range of services to further this aim such as transitional employment, supported employment, and services for members in independent employment. Transitional employment is a form of employment support provided in the Clubhouse model (C. McKay et al., 2016) and offers employment for members in the open labour market for a limited period of 6–9 months, earning at least the minimum wage. Transitional employment is administered by the Clubhouse but is evaluated and paid by the employer (Clubhouse International, 2018, p. 22).

Although the Clubhouse model emphasizes providing support for members to gain and maintain paid employment, the standards outline further support functions of the Clubhouse in the sections of education and functions. These include transportation, community support services, advisory functions, affordable housing, and promoting and maintaining a healthy lifestyle (Clubhouse International, 2018, pp. 27–28); each of these addresses at least one of the multifaceted problems raised by mental illness (Propst, 1997). Social programs are also offered at Clubhouses (Clubhouse International, 2018, p. 29). Propst (1997) described the social programs as having a dual purpose: (1) they help those who are otherwise occupied during the WOD to keep in touch with the community, and (2) they reinforce the relationships formed by working together during the WOD.

The remaining two domains of space, and governance and funding of the standards prescribe the administrative requirements for each Clubhouse. Interestingly, they require member participation at all levels of Clubhouse representation and decision-making (Clubhouse International, 2018, p. 33 & 37), thereby highlighting the equal nature of the program.

## The Clubhouse model as the basis of PSR

Although developed gradually, the Clubhouse standards define a practice that both reflects the principles of PSR and plays an important role in developing these principles (Anthony & Liberman, 1986; Cnaan et al., 1988). Cnaan et al. (1988) derived the general principles of PSR by analysing the Clubhouse model and the practice of Horizon House, another pioneer in the field. Beyond their main difference, which is that the Clubhouse offers lifelong support whereas Horizon House focuses on helping people return to independent living as soon as possible, Cnaan et al. (1988) identified 13 basic principles of PSR, which have been confirmed by later studies (Anthony et al., 1999; Anthony & Mizock, 2014; Farkas et al., 2007). According to these principles, PSR is based on the understanding that, despite their diagnosis, all people can improve their life by bringing it closer to what is ordinary in the community if they are provided with the necessary skills (Cnaan et al., 1988; Rössler, 2006).

PSR has a social rather than a medical focus by shifting attention from a person’s illness to the person’s abilities (Farkas, 2006; Rössler, 2006). In addition, PSR recognizes that every individual has different needs, which may require different kinds of support, and that individuals with mental illness are capable of making competent decisions about the interventions they need for their own recovery (Rössler, 2006). Staff should support by being fully committed to the principles of PSR and should form a relationship beyond a professional façade with people using PSR services (Cnaan et al., 1988; Kidd et al., 2017). It is also the individual’s decision about whether to join a PSR program, which should be easy because of the few inclusion criteria for PSR services and the requirement of physical accessibility (Anthony & Liberman, 1986; Cnaan et al., 1988). All PSR interventions should concern not just the person’s immediate environment but should also aim for necessary changes on a broader, societal level (Rössler, 2006) and be prepared to provide early intervention in cases of relapse (Cnaan et al., 1988). Work is central in PSR because participation in meaningful work and aspiring for gainful employment are restorative and reintegrative given the wide acceptance of the worker role in society (Farkas & Anthony, 2010). However, this work focus shifted slightly in later stages of the development of the field towards programs helping people to cope with symptoms and supporting families in caring for their relatives affected by mental illness (Anthony & Liberman, 1986; Farkas et al., 2007).

## The Clubhouse Model in the context of salutogenic theory

A common ground for salutogenesis and the PSR field, including the Clubhouse model, is the principle of being concerned with human beings as a whole and not reducing them to an illness (A. Antonovsky, 1979; Farkas & Anthony, 2010). Salutogenesis, PSR, and the Clubhouse model all acknowledge that the environment is also important in terms of the outcome (A. Antonovsky, 1979, 1987; Cnaan et al., 1988; Farkas, 2006; Farkas & Anthony, 2010; Farkas et al., 2007). Outcome orientation, self-determination, and empowerment are also values shared between salutogenesis, PSR, and the Clubhouse model (A. Antonovsky, 1987; Cnaan et al., 1988; Farkas & Anthony, 2010; Rössler, 2006).

### Basic assumptions

Considering the basic assumptions of salutogenesis reveals further similarities with the Clubhouse model. What follows is a systematic examination of how elements of the Clubhouse model can be understood in the context of each assumption.

#### Health as a continuum

The first basic assumption of salutogenesis is the notion of health as a continuum, which means to “study the location of each person, at any time, on this continuum” (A. Antonovsky, 1987, p. 2). Assessing the status of a person’s well-being assumes that all people are healthy to a certain degree despite the presence of illness; consequently, healthy aspects become apparent in addition to those affected by illness (Vinje et al., 2017). Similarly, the Clubhouse model acknowledges the capability of members by entrusting them with the responsibility for the operation of the Clubhouse (Clubhouse International, 2018, p. 11). Tanaka and Davidson (2015b) observed that all members are treated as being contributors to the community independent of their level of participation in everyday tasks. These attitudes are likely to help to move a person or group to a higher level on the continuum.

#### Story of the person

Antonovsky encouraged a holistic approach to well-being rather than a sole focus on disease; this was not just for compassionate reasons but to be able to see the complex context of a person’s health status (A. Antonovsky, 1987). This concept is emphasized in the language used by both the salutogenesis and Clubhouse models. Salutogenesis uses the term “person” to describe the individual in focus (Vinje et al., 2017). Similarly, the Clubhouse terminology uses the term “member” instead of “patient” throughout the complete standards document (Clubhouse International, 2018). This approach seems to be more than a choice of words because, although the term “patient” indicates a person defined by illness, the term “member” infers the positive notion of being a contributing member of a community. This notion focuses attention on the person’s environment as an important component of well-being, which is also a major element of the PSR ecological approach (Anthony & Liberman, 1986; Farkas, 2006; Rössler, 2006).

#### Health-promoting (salutary) factors in focus

Because risk factors are omnipresent, salutogenesis assumes a positive approach to health by shifting the focus from pathogens to salutary factors (Langeland et al., 2007). In this sense, the aim is not to treat a disease but to build on one’s available resources to be able to improve (A. Antonovsky, 1979, 1987). Similarly, the Clubhouse model has a strength-based approach and explicitly separates itself from the illness-focused mental health system (Clubhouse International, 2018, pp. 15–16). This parallels the principle of PSR, which postulates that all people can improve their level of functioning (Cnaan et al., 1988).

#### Tension and strain as potentially health promoting

The fourth basic assumption of salutogenesis is the consideration of tension and strain as potentially health promoting (Vinje et al., 2017). From the salutogenic perspective in which stressors are omnipresent and tension is general, continuous coping is crucial. However, A. Antonovsky (1979) proposed that salutogenic coping with tension is a positive experience because it has a positive impact on people’s well-being by improving the SOC and thus helping them to develop the necessary coping skills. Therefore, tension is viewed as a prerequisite for personal growth to achieve a more constructive and stronger identity (Langeland et al., 2016; Magrin et al., 2006). However, the tension required to cope must pose an appropriate challenge by being neither too easy nor too difficult or, in salutogenic terms, it must have an “underload/overload balance” (Idan et al., 2016). Therefore, appropriate challenges are important (Langeland et al., 2007) and might be defined as “the salt of life” (Magrin et al., 2006).

The major challenge for Clubhouse members is taking responsibility. At the same time, the model’s approach to member responsibility seems to be ambiguous. Although members are given challenges in the Clubhouse model, at the same time they are protected from them (Clubhouse International, 2018, pp. 3, 9, 11 & 16). In particular, two standards promote taking responsibility by stipulating that Clubhouses cannot be run without member involvement and members should have shared responsibility in operating the Clubhouse. However, other standards prohibit practices that enforce members’ participation by paying or rewarding members for their contribution and by limiting Clubhouse work to those tasks generated by the Clubhouse, which arguably eliminates the competitiveness of regular working life. The PSR literature (Anthony & Liberman, 1986; Rössler, 2006) notes that an important aspect of PSR is reducing stress on the individual. However, in the context of salutogenesis, limited buffering of stressors is needed to provide appropriate, skill-building challenges to increase well-being.

#### Active adaptation

According to the fifth basic assumption of salutogenic thinking, treatment must reflect that the individual is in a constant process of learning and change because of the need to face and meet challenges. Therefore, active adaptation during treatment is necessary to increase a person’s salutogenic capacity (Langeland et al., 2007) instead of assuming a “right treatment based on the right diagnosis” approach. In broad terms, salutogenesis considers any intervention that leads to improvement as treatment.

It is not apparent whether the Clubhouse model shares the principle concerning active adaptation as the ideal in treatment. On the one hand, we consider it to be a personalized treatment that members can decide whether and how they wish to use the Clubhouse (Clubhouse International, 2018, pp. 3 & 20). On the other hand, a condition within the principle of active adaptation is that it is necessary for finding an ideal treatment. It is not clear from the standards whether there is any procedure in place to ensure that members use the Clubhouse in a way that would be ideal for them or whether using the Clubhouse has a positive effect on their life. However, some studies (Macias et al., 1999; Raeburn et al., 2013) report that case management is offered by several Clubhouses, and such a service would likely involve evaluating “treatment” success.

### SOC in the Clubhouse model

As the central concept in salutogenic theory, SOC offers the key to coping with stress and helping people maintain an optimal position on the health continuum (A. Antonovsky, 1979). The level of SOC is determined by its three core components: comprehensibility, manageability, and meaningfulness. A higher level of SOC indicates a higher level of well-being (Vinje et al., 2017), and a strong SOC is claimed to be universally beneficial to all people (Landsverk & Kane, 1998). Similarly, the Clubhouse model aims to work towards improving members’ well-being (Clubhouse International, 2018).

#### Comprehensibility

Comprehensibility is the cognitive component of SOC and is described as experiencing stimuli as ordered instead of chaotic, consistent rather than irrational, structured instead of random, and clear rather than ambiguous (Vinje et al., 2017). Correspondingly, the Clubhouse aims to introduce structure—not unlike what usually happens in society—to members’ lives by being open on weekdays during business hours (Clubhouse International, 2018, p. 17). In addition, the standards themselves help to promote consistency and clarity by explicitly providing a definition of the model.

#### Manageability

The second component of SOC, manageability, refers to the ability to manage stimuli if resources are available to meet the demands. Notably, stimuli can be derived from one’s internal and external environments (Vinje et al., 2017). According to the preamble to the standards (Clubhouse International, 2018), Clubhouses offer members help in tackling the challenges of life by claiming their goal to be “helping people with mental illness to stay out of hospitals while achieving social, financial, educational and vocational goals.” Arguably, offering this help to members might qualify as a factor supporting one’s sense of manageability.

#### Meaningfulness

The last component of SOC, meaningfulness, is the most important SOC factor according to A. Antonovsky (1987) because it is an emotional–motivational entity that determines whether something matters enough for the individual to deal with, and thus plays a crucial role in shaping the outcome of coping. To determine whether the Clubhouse model supports meaningfulness, we studied its crucial areas.

#### Main activity

We had positive findings about the main activity because Clubhouses offer regular, engaging, and meaningful activities for members (Clubhouse International, 2018, pp. 17–18). In particular, the standards stipulate that Clubhouses must stay open during regular business hours and that work activities must be engaging and meaningful. This also helps members to structure their lives similarly to societal practice, which is consistent with the “normalization” principle of PSR (Cnaan et al., 1988; Farkas, 2006).

#### Existential issues

Existential issues in salutogenic terms refer to being able to form a view of life in ideological, political, and/or religious terms (Lindstrøm, as cited in Vinje et al., 2017, p. 28). In this sense, having hope and/or a stable belief system might be as important for the individual as building up a logical, solid, and consistent lifeworld. We examined whether the Clubhouse model has standards to establish a rule of law in the Clubhouse community and found several examples. First, the preamble establishes the status of the standards as “a ‘bill of rights’ for members and a code of ethics for staff, board and administrators.” Furthermore, several standards outline members’ rights as having full and equal access to all facilities and giving them authority over anything that might personally concern them (Clubhouse International, 2018, pp. 5, 8 & 14). In addition, the standards stipulate members’ rights to dignity and respect, and establish an equal hierarchy between members and staff. These elements, together with the stipulation of unlimited membership (Clubhouse International, 2018, p. 1), offer stability and security, which are important existential aspects. However, the Clubhouse standards do not contain any specific measures for life outside the Clubhouse community. Nevertheless, it is reasonable to assume that having access to a supportive community and gaining increased self-confidence would have a positive effect on members’ lives outside the Clubhouse. Similarly, the standards do not contain explicit reference to increasing or maintaining hope, an important aspect of the recovery process (see, for example, Anthony & Mizock, 2014) and do not explicitly mention developing a stable belief system. Nonetheless, the conclusion about the support function of belonging to and being able to rely on a community might also be true for these elements.

#### Inner feelings

Inner feelings can indicate a stable state of mind and an awareness of one’s emotions, although it is difficult to separate these general categories from particular emotions that are evoked when a person interacts with his or her environment. However, considering individual aspects only, we did not find explicit examples to indicate that the Clubhouse model contributes to the emotional self-awareness or mental stability of members. Nevertheless, it is again a reasonable supposition that elements such as the principles of self-determination, recovering at one’s own pace, conveying respect, and providing a supportive community constitute implicit considerations of members’ emotions.

#### Social relationships

Although the standards do not explicitly mention individual members’ inner feelings, several ingredients of the model seem to focus on social relationships and building a community, which arguably have a bearing on a person’s social life (Clubhouse International, 2018, pp. 1, 6–7, 18 & 32). These standards seem to focus on three main topics: membership and personal security, keeping members in the community, and strengthening the community by fostering relationships.

### Resistance resources (RRs) in the Clubhouse model

As noted, RRs can be general (GRR) or specific (SRR) (Mittelmark et al., 2016). We defined a GRR as a consistent life experience of general utility that is instrumental in shaping the outcome of the coping process. We defined SRR as a tool for coping with a particular situation. With regard to the distinction between GRRs and SRRs, we considered general Clubhouse opportunities to be available universally for everyone as GRRs, and specific support used by individual members as SRRs. We then classified the opportunities described in the standards detailing the available Clubhouse services, such as employment and education support, community support services, transportation and housing services, assistance with a healthy lifestyle, and social programs as GRRs (Clubhouse International, 2018). We considered specific uses of these services by a member in a way that is helpful to their particular problem to be SRRs. For example, the Clubhouse offers help with housing problems, which we took to be a GRR, but explicitly offering housing to members or helping them to benefit from a housing program was considered to be an SRR.

**Figure 2.**
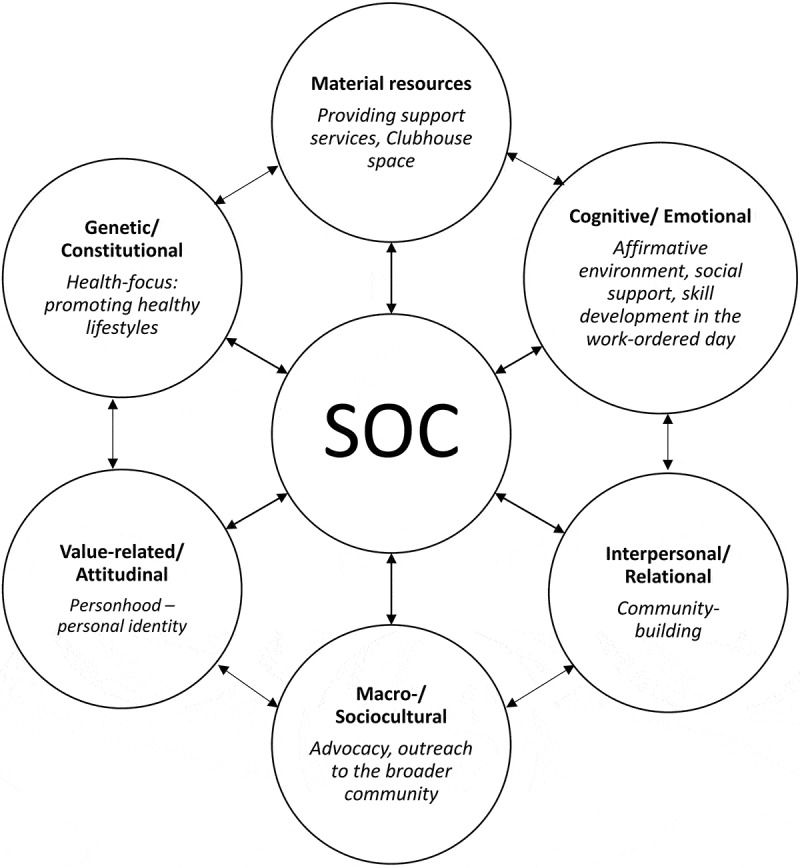
The hypothetical interaction between members’ sense of coherence (SOC) and use of different resistance resources (RRs) in the Clubhouse setting

According to the RR–RD continuum model, the type of life experience can serve as the basis for the classification of RRs, as well as their counterparts, the general and specific resistance deficits (RDs). A summary of some of the types of RRs and RDs is presented in Figure 1. Based on this typology, we suggest in Figure 2 a classification of some of the major Clubhouse interventions. Note that there is a dynamic and reciprocal relationship between SOC, RRs, and RDs, and between RRs and RDs themselves (Idan et al., 2016). Although each type of RR or RD would, respectively, strengthen or weaken the SOC, the SOC would also have an effect; that is, a strong SOC would likely help in acquiring and using RRs, whereas a weak SOC would act in the opposite manner. In addition, a similar interplay may also be assumed between the different groups of RRs because the presence of one might promote the use of another or the absence of one might hinder the use of another.

Constitutional resources are a person’s physical and biochemical disposition to cope with problems (A. Antonovsky, 1979). Therefore, one goal of Clubhouses—to support their members with a healthy lifestyle (Clubhouse International, 2018, p. 28) by helping to improve their physical health as well as their mental well-being—could be considered a resource. Material resources are the means to sustain life both in a biological and social sense; therefore, any services provided by a Clubhouse such as providing support to improve physical and/or mental well-being can be considered as RRs. For example, such a service could be the Clubhouse itself as a place to provide support or the employment support provided by the Clubhouse. The next group of resources concerns cognitive abilities, such as the intelligence to obtain knowledge and the possession of necessary knowledge, as well as emotional aspects such as a strong, stable sense of self (identity), which are crucial to tackling challenges. The model ingredients relevant here are those that help with the comprehensibility factor of the SOC.

Another crucial type of RR is having high-quality social relationships and a sense of belonging in the community, both of which seem to be a priority of the model according to the standards. On another level, this translates into the fifth group of macro or sociocultural factors, such as being part of a stable society that does not require constant adaptation by an individual in response to changing conditions. Finally, a crucial source of coping resources includes attitudes and values, such as a preventive attitude towards problem-solving and a stable belief system in which to operate. In the Clubhouse model, these terms are partly covered by the ingredients that support the existential issues described above and the status as “members” of a Clubhouse.

## Discussion

In this article, we analysed the Clubhouse model in the context of the theory of salutogenesis to address claims that a comprehensive theory was lacking to inform research on the Clubhouse model (Mowbray et al., 2006; Mutschler et al., 2018; Raeburn et al., 2015). We have suggested that using salutogenesis as a theoretical framework for the model might also provide a foundation for its application in PSR research because research in these two fields faces similar challenges. For instance, diversity is an important challenge, particularly in terms of interventions, user groups, and goals, which make it difficult to achieve sample sizes large enough to make comparisons (Farkas et al., 2007; Rössler, 2006).

The starting point of this paper was that as a broad, established theory, salutogenesis might provide a unifying platform to address these issues. In addition, by providing insight into how well-being is achieved by strengthening the SOC, we reasoned that the SOC questionnaire (A. Antonovsky, 1987) may provide a useful tool for measuring outcomes. Therefore, we conducted a systematic examination to identify the different elements of the Clubhouse model, with an emphasis on those prescribed by the standards, and how these can be understood in the context of salutogenesis.

We found that Clubhouse practices such as a PSR intervention are highly consistent with salutogenic principles, and we suggest that salutogenesis might be a suitable theoretical framework for the Clubhouse model. However, we also identified areas where salutogenic theory might help improve the Clubhouse model, and vice versa.

For example, assuming that reclaiming the role of an equal participant in society (outside the Clubhouse model) is one of the major aims of a person’s PSR and one of the foremost ways salutogenesis might improve Clubhouse practices (Magrin et al., 2006). This means acknowledging and implementing the concept of appropriate challenge as a health-promoting factor. Based on the standards, the current practice is ambiguous in this matter. On the one hand, members are expected to contribute to the operating of a Clubhouse (Clubhouse International, 2018, pp. 9 & 11). On the other hand, the standards offer lifelong membership regardless of an individual’s contribution (Clubhouse International, 2018), and empirical studies show that Clubhouse members are likely to choose to remain within the safety of the Clubhouse community rather than cope with the challenge of leaving it. For instance, Raeburn et al. (2013) identified the potential risk of developing “service dependence” for Clubhouse members. The results of a recent meta-synthesis of 11 qualitative studies (Kinn et al., 2018b) drew a similar conclusion. According to this study, the Clubhouse community encourages members in the processes of “pushing their boats out” onto the “open sea” of society and work life, after a supportive and building-up period of being “anchored” in the Clubhouse (Kinn et al., 2018b). However, despite flourishing when participating in Clubhouse activities, some members felt they were not ready to leave the “safe harbor” for fear of the challenges outside the Clubhouse. We suggest that consciously offering greater challenges that members can relate to real-life problems may help their social integration as part of their recovery (Kinn et al., 2018b).

Next, active adaptation as the ideal in treatment is another area for possible development. A person’s active adaptation in a treatment means using the appropriate measure to solve a given problem (Griffiths, 2009; Vinje et al., 2017). Therefore, to select an appropriate measure, one must assess the given situation. Notably, although it is possible for people to tailor their use of the Clubhouse to their individual needs, we could not determine from our review of the standards whether a recovery plan is developed for each person, and we conclude that it may not be an integral part of the model. The absence of such a plan suggests that there is no kind of assessment in place, which makes it impossible to change the course of intervention (tailoring), which is a postulate of the active adaptation concept. We suggest that such a systematic approach to member support may result in a more effective intervention.

Salutogenesis emphasizes the importance of personal relationships for strengthening the meaningfulness component and thus the SOC. Although the community is a definite focus of the model, we found that the standards lack elements concerning individual relationships within the community and, therefore, the initiatives directly concerning the members’ inner feelings. Moreover, except for helping members to complete tasks and become employed or educated in society, there seem to be no Clubhouse initiatives to help the individual members integrate into society and to improve their well-being outside the Clubhouse. This was also evident from the empirical studies on individual and social relationships in the Clubhouse model. For instance, although some studies (Carolan et al., 2011; Fekete et al., 2020) found that the Clubhouse model provides opportunities for rebuilding social networks, a sense of belonging, and contact with peers, another study (Pernice-Duca, 2008) claimed that fellow members were least likely to be nominated as a source of support within Clubhouse members’ social networks. Similarly, Biegel et al. (2013) argued that, whereas members perceive their Clubhouse as a place to belong and a family substitute, they listed remarkably few individuals from their Clubhouse environment as part of their social networks. Furthermore, a recent Norwegian study did not find a significant correlation between members’ loneliness and use of a Clubhouse (Bonsaksen et al., 2019). On the other hand, several studies have shown that Clubhouse members experience a close emotional connection with their Clubhouse environment. (Biegel et al., 2013; Fekete et al., 2020; Pernice-Duca, 2008; Schiff et al., 2008). In addition, other studies suggest that members experience the Clubhouse environment as supportive, affirmative, and accepting (Norman; Schiff et al., 2008). In summary, we suggest that, although there is a development potential in terms of individual relationships within the Clubhouse model, it may be based on the foundations of a strong community reported in empirical studies.

One of our major findings was that the services and opportunities offered by Clubhouses can be understood as RRs, and we differentiated between GRRs and SRRs according to the range of their utility (Mittelmark et al., 2016). In this article, we propose a classification of general Clubhouse services according to the classes of GRRs discussed in the salutogenic literature (A. Antonovsky, 1987; Langeland et al., 2007; Sullivan, 1989). We suggest that the particular solutions a member might use within these general opportunities could be considered as SRRs. Even though this approach seems to be operational and logical in our case, it is arguably ambiguous because, with the same utility-range definition, several other logical models can be outlined. For example, from a macroperspective, mental health care could be a GRR within the universal health-care system. In this sense, the Clubhouse model as an intervention within the mental health services could serve as an SRR. Therefore, we suggest that improvement and concretization of the definition of GRRs and SRRs are necessary. Arguably, the expertise of PSR with factors that facilitate a person’s rehabilitation process might be helpful in the further development of the RR concepts.

Lastly, we emphasize the potential benefits of the common positive terminology in the salutogenesis and Clubhouse models as a matter for consideration. Salutogenesis focuses on seeing the person behind the diagnosis and considers tension as an ordinary occurrence in everyday life. The use of the word “person” in salutogenesis and reference to Clubhouse participants as members illustrate the transition from “patienthood to personhood” (Peckoff, 1992) through which the person becomes a contributing community member, which is a positive step from being considered as a “mental health patient.” We suggest that, from a societal perspective, promoting this approach may also help to reduce the stigma and prejudice faced by someone who is labelled as a “patient” or “service user,” and instead referring to them by neutral terms such as “member” or “person”, which makes these people like everyone else in society, who all must learn to cope with tensions and challenges in their lives.

## Conclusion and limitations

In the present paper, we have explored the Clubhouse model in the salutogenic context by discussing the different salutogenic factors and RRs contained in the Clubhouse model. In conclusion, this study suggests that salutogenesis seems to be a suitable framework for Clubhouse research and practice, and for PSR as well.

Given the considerable insight of salutogenesis into how well-being is achieved, we suggest that Clubhouses might play a role in promoting a stronger SOC, which is the foremost agent in promoting mental health and well-being. As we proposed in our preliminary classification, Clubhouse interventions aiming to achieve well-being may function as GRRs (or SRRs). Further studies of these interventions and the insight of PSR into factors that assist an individual’s rehabilitation process may help in the further development of the RR concepts of salutogenesis.

Admittedly, this paper is only a theoretical elaboration and, therefore, constitutes a string of ideas for answering salient questions about PSR, such as suggestions for future research and practical development, using the Clubhouse model as an example. Consequently, we emphasize that, although we have presented some ideas and findings, further research is needed to determine whether they have empirical merit. The next step is to perform empirical studies of the Clubhouse model from the salutogenic perspective.
